# Global optimization and oxygen dissociation on polyicosahedral Ag_32_Cu_6_ core-shell cluster for alkaline fuel cells

**DOI:** 10.1038/srep11984

**Published:** 2015-07-07

**Authors:** N. Zhang, F. Y. Chen, X.Q. Wu

**Affiliations:** 1 State Key Laboratory of Solidification Processing, Northwestern Polytechnical University, Xian 710072, China

## Abstract

The structure of 38 atoms Ag-Cu cluster is studied by using a combination of a genetic algorithm global optimization technique and density functional theory (DFT) calculations. It is demonstrated that the truncated octahedral (TO) Ag_32_Cu_6_ core-shell cluster is less stable than the polyicosahedral (pIh) Ag_32_Cu_6_ core-shell cluster from the atomistic models and the DFT calculation shows an agreeable result, so the newfound pIh Ag_32_Cu_6_ core-shell cluster is further investigated for potential application for O_2_ dissociation in oxygen reduction reaction (ORR). The activation energy barrier for the O_2_ dissociation on pIh Ag_32_Cu_6_ core-shell cluster is 0.715 eV, where the *d*-band center is −3.395 eV and the density of states at the Fermi energy level is maximal for the favorable absorption site, indicating that the catalytic activity is attributed to a maximal charge transfer between an oxygen molecule and the pIh Ag_32_Cu_6_ core-shell cluster. This work revises the earlier idea that Ag_32_Cu_6_ core-shell nanoparticles are not suitable as ORR catalysts and confirms that Ag-Cu nanoalloy is a potential candidate to substitute noble Pt-based catalyst in alkaline fuel cells.

Alkaline fuel cells have advantages of using non-platinum metals as electrode catalysts, because alkaline solution is less corrosive than acidic media. For fuel cells in alkaline media, hydrogen or metal is oxidized at the anode, releasing electrons. Meanwhile, oxygen is reduced at the cathode, producing hydroxide ions. A much wider range of metals are stable in alkaline environments, including less expensive materials such as nickel and silver. The combination of reasonably high activity, good long-term stability, and relatively low price (~2% the price of Pt) make Ag attractive as an alkaline oxygen reduction reaction (ORR) electrode catalysts. Hence, silver is regarded as the most promising metal to replace platinum^1^,^2^.

The oxygen reduction reaction (ORR) in alkaline media is of great industrial importance. The ORR serves as the cathode reaction in alkaline fuel cells^3^,^4^,^5^ and metal-air batteries^6^,^7^,^8^. Fuel cells present the possibility of new green power source with high efficiency energy conversion. However, the sluggish kinetic continues to limit oxygen cathode performance, and the resulting large cathode overpotential leads to lower energy efficiency and lower power density.

Oxygen reduction in aqueous alkaline media is a complicated electrocatalytic reaction. The oxygen dissociation on the surface of catalyst is an initial step and is important for determining activity level of the ORR reaction^9^. Previous studies show that redox property of the silver catalyst is a controlling factor of the ORR activity which is affected by silver surface structure and chemical state. For instance, a relatively higher affinity of Ag(110) for O_2_ leads to a higher ORR activity^10^. Alloying is a general technique to improve ORR activity and the stability of catalysts. It has been reported that alloying Ag with Mg produces a catalyst with better ORR activity than Pt^11^. Recent theoretical works have been focused on nanoscale system, typically, core-shell or alloy Ag_13-x_Cu_x_ and Ag_38-x_Cu_x_ clusters. The alloyed cluster of truncated octahedron (TO) Ag_32_Cu_6_ shows a better catalytic activity than the TO Ag_32_Cu_6_ core-shell cluster^12^. More recent theoretical calculations using a periodic slab show that alloys of Ag with the late 3*d* transition metals (Fe,Co,Ni,Cu) bind the OOH intermediate more strongly than pure Ag, thereby accelerate the rate-limiting step^13^.

However, the determination of the most stable structure of nanoparticle^13^ is a crucial step for understanding their ORR property and designing possible industrial application^14^. Due to the lack of translational invariance constraints, nanoparticles can assume a much wide variety of geometric structures than bulk crystals. This is the origin of their very complex energy landscapes^15^, which requires *ad hoc* computational tools in order to be efficiently explored, so that low-energy configurations can be singled out. The situation is even more difficult for binary nanoparticles^16^. In fact, binary nanoparticles not only present a wide variety of geometric structures, but also different types of chemical ordering, *i.e.* of the pattern in which the two atomic species are arranged within the geometric structures. The algorithms that have been developed to deal with the problem of finding low energy-structures are known as global optimization algorithms.

For this reason, we perform the global optimization of bimetallic Ag–Cu clusters within genetic algorithms (GA). An atomistic model of the 38-atom Ag-Cu system is developed firstly through local optimization search within the Gupta model. The global optimization searches are used to build up a large data base of structures, comprising as many structural motifs as possible. Finally, the best clusters of each motif are locally optimized at the density functional theory (DFT) level. It is demonstrated that, the TO Ag_32_Cu_6_ core-shell cluster is less stable than the polyicosahedral (pIh) Ag_32_Cu_6_ core-shell cluster from both atomistic models and DFT calculations. And the newfound pIh Ag_32_Cu_6_ core-shell cluster is further investigated to dissociate O_2_ in oxygen reduction reaction (ORR).

## Results and Discussion

### Cluster evolution

**Figure 1:**
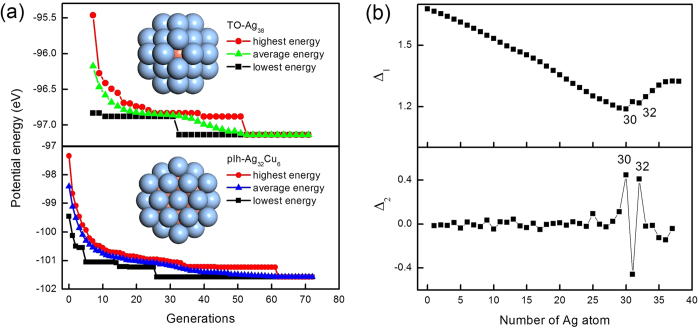
(**a**) Energy evolution progress plot in GA program for the truncated octahedron Ag_38_ cluster and the polyicosahedral Ag_32_Cu_6_ core-shell cluster. Symbols: ▲ average energy, ■ lowest energy, ● highest energy. (**b**) Δ_1_ and Δ_2_ in binding energy for the 38 atom Ag-Cu cluster system.

Evolutionary progress plots during the convergence of the GA on the lowest energy Ag_38_ and Ag_32_Cu_6_ cluster are shown in Fig. 1(a). The figure shows that the GA requires 74 generations to find the lowest energy structure of Ag_32_Cu_6_ cluster but only 35 generations to find the lowest energy structure of Ag_38_ clusters. In both cases, the energy (highest, lowest and average - the average value of its total potential energy for the population) are becoming equal with increasing generation number, indicating that the population converges on a single structure.

These results are typical of the other cluster nuclearity studied. Binary nanoparticles present a wide variety of geometric structures and different types of chemical ordering. And the potential energy surface is therefore likely to have more local minima for GA to search, leading to greater difficulty in finding the global minimum. It should be noted that the population convergence does not always signify that the GA has found the true global minimum. Population convergence corresponds to a loss of population diversity, and the diversity can be reintroduced into a population either by mutation or by crossover in the GA, so the converged structure is found to be the global minimum in each case.

### Structural stability

In order to compare the relative stability of clusters of different compositions, we monitor the quantities Δ_1_ and Δ_2_ adapted to binary clusters. Δ_1_ is the excess energy with respect to *N* bulk atoms divided by *N*^2/3^ where as the second difference (Δ_2_) in the energy describes the relative stability of the cluster as compared to the neighboring compositions. Δ_1_ and Δ_2_ are defined as:


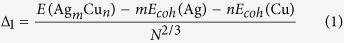



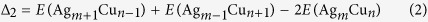


where *N* = *m* + *n* and *E*(Ag_*m*_Cu_*n*_) is the global-minimum energy of the Ag_*m*_Cu_*n*_ clusters. *E*_*coh*_(Ag) and *E*_*coh*_(Cu) are the cohesive energies per atom of the bulk metals Ag and Cu, respectively. Stable structures are identified by low Δ_1_ value. Maxima of Δ_2_ indicates structures of special relative stability which is compared to those of the same size and nearby compositions.

**Figure 2:**
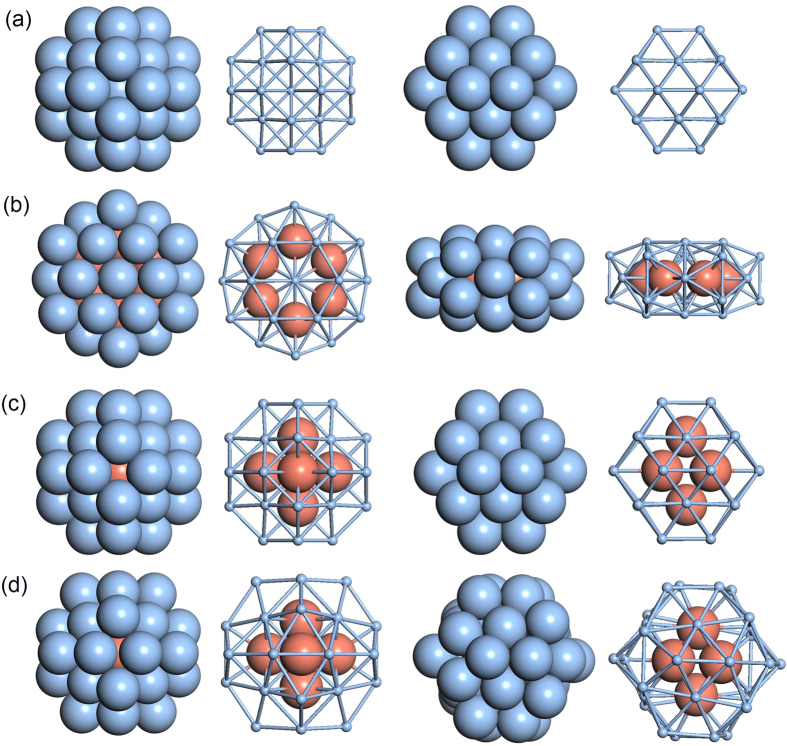
Global optimization structure found by GA at the atomistic potential level: (**a**) The bulk-like truncated octahedron cluster for Ag_38,_ (**b**) the polyicosahedral core-shell cluster for Ag_32_Cu_6_ and (**c**) the TO core-shell isomer for Ag_32_Cu_6_. (**d**) Low energy structure for TO Ag_32_Cu_6_ core-shell cluster after DFT local reoptimization. Each structure is shown in two views with two styles. Ag atoms are reprented in blue and Cu atoms are reprented in light red.

Figure 1(b) compares the Δ_1_ and Δ_2_ for the lowest-energy 38-atom clusters with different Ag atom numbers, from which we can single out the especially stable composition at n = 30 and 32 Ag atoms. For these clusters, the minima in Δ_1_ and maxima in Δ_2_ are found to concur. These locally stable structures are Ag_30_Cu_8_ and Ag_32_Cu_6_ clusters. As shown in Fig. 2, Ag_32_Cu_6_ cluster has the perfect core-shell pIh structure where Cu atoms forming a ring, and is completely covered by a single layer thick Ag shell. Morever, the Ag_32_Cu_6_ cluster has the highest D_6h_ symmetry of the whole sequence of the heterogeneous clusters. Ag_30_Cu_8_ cluster, as shown in Figure S1, is a disordered core-shell structure with a low symmetry of C_s_ which includes the maximum number of small atoms inside, that is to say, 8-Cu-atom core is embedded in a 30-Ag-atom shell. Thus, Ag_32_Cu_6_ with D_6h_ symmetry is a magic core-shell cluster for the 38-atom Ag-Cu clusters. Liking other magic clusters, the pIh-Ag_32_Cu_6_ cluster has several common features, i.e., a high-symmetry core-shell atomic order and complete geometry shape.

As shown in Fig. 2, the Gupta model predicts that the global optimization structure as found by GA for Ag_38_ is the perfect TO, and the global optimization structure for Ag_32_Cu_6_ is the pIh core-shell cluster where an isomer for Ag_32_Cu_6_ is the TO core-shell cluster.

**Figure 3:**
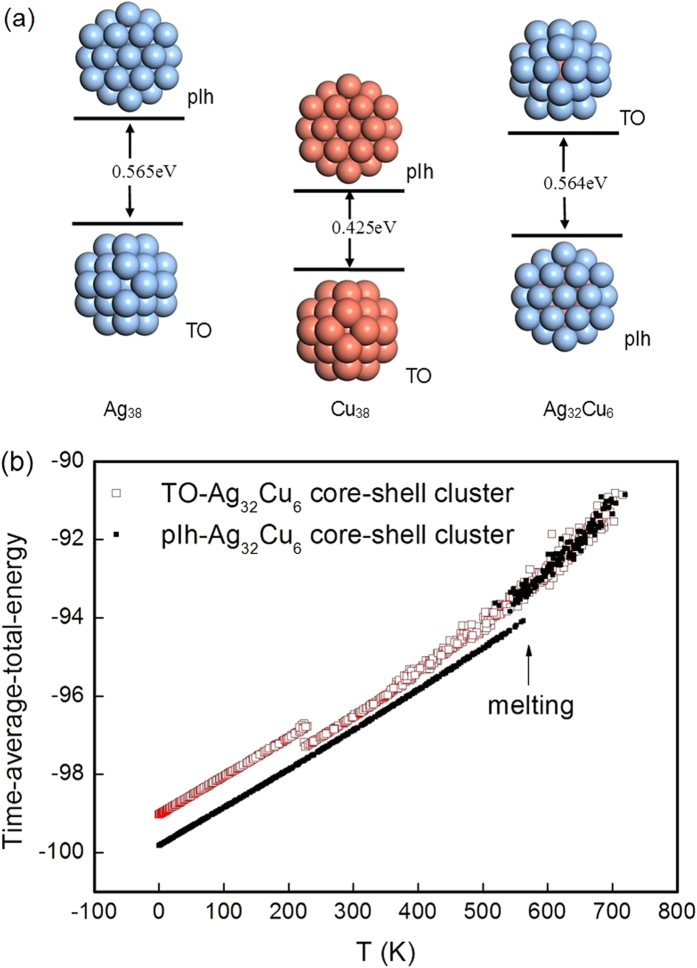
(**a**) DFT-level energy difference between the truncated octahedron and the polyicosahedral structures for Ag_38_, Cu_38_ and Ag_32_Cu_6_ clusters. (**b**) Caloric curves at the atomistic potential level for the truncated octahedron and the polyicosahedralAg_32_Cu_6_ core-shell clusters.

A further comparison between atomistic and DFT results is significant for checking the reliability of the atomistic potential in producing the pIh and TO core-shell clusters. Figure 3(a) shows the total energy difference between the TO and the pIh structures for Ag_38_, Cu_38_ and Ag_32_Cu_6_ clusters obtained from the DFT optimization. It is shown that there are different levels of structural stability among the isomers of 38-atom clusters. In Ag_38_ nanocluster, the total energy of the TO structure is 0.565 eV less than the pIh structure, and similarly in the Cu_38_ nanoclusters, the total energy of the TO structure is 0.425 eV less than that of the pIh structure. The total energy of the TO structure is 0.425 eV less than that of the pIh structure. The energy difference between the TO and pIh structures in pure Cu_38_ is smaller than the corresponding energy difference in pure Ag_38_. These results indicate that the order of stability is TO > pIh. The findings are consistent with a previous report^17^. However, the Ag_32_Cu_6_ core-shell clusters where all the Ag atoms are on the surface and all Cu atoms are inside show that the total energy of the pIh structure is 0.564 eV less than that of the TO structure. The TO Ag_32_Cu_6_ core-shell cluster has both (100) and (111) facets in the surface layer, and the pIh Ag_32_Cu_6_ core-shell structure has only (111) facets in the surface layer. During the DFT optimization, as shown in Fig. 2(d), the TO Ag_32_Cu_6_ core-shell cluster shows instability and the surface layers can no longer be identified as (100) and (111) facets, so only the pIh Ag_32_Cu_6_ core-shell structure is further investigated for its catalytic characteristics. It is a newfound structural stability for 38-atom Ag-Cu bimetallic geometry. Previous work shows that the energy of TO Ag-Cu core-shell cluster is lower than TO Ag-Cu alloyed cluster^18^. We evaluate the catalytic performance of the pIh Ag_32_Cu_6_ core-shell structure because of its great academic importance.

Figure 3(b) compares the caloric curves for pIh and TO Ag_32_Cu_6_ core-shell clusters. For the pIh Ag_32_Cu_6_ core-shell cluster, the solid-liquid transition takes place in the temperature range of 525–600 K, which can be identified from the changes in the slope of the caloric curves. We have repeated the melting study of TO Ag_32_Cu_6_ core-shell cluster, which is not a global minimum structure. In this case, the cluster undergoes a solid-solid transition from the TO to the amorphous structural motif before melting occurs. The caloric curves show that, the total energy of the TO Ag_32_Cu_6_ core-shell cluster is slightly higher than that of the pIh Ag_32_Cu_6_ core-shell cluster at low temperatures, which indicating that TO cluster is a metastable isomer. Approaching 220 K, the TO cluster sharply transforms to the amorphous cluster. At higher temperatures, the caloric curves for the two Ag_32_Cu_6_ core-shell clusters are the same, indicating that the TO Ag_32_Cu_6_ core-shell cluster is unstable and its transition from order to disorder is thermodynamically favorable.

### O_2_ direct dissociation on Ag_32_Cu_6_ nanoparticles

Oxygen reduction in aqueous alkaline media is a complicated electrocatalytic reaction. Many species have been proposed as intermediates in this multistep reaction, including O, OH, O_2_ and OOH. The four-electron reaction of the oxygen reduction reaction (ORR) from O_2_ reduction to OH^−^ in alkaline environment is: O_2_+2H_2_O+4e^−^→4OH^−^. There are two four-electron pathways as shown in Scheme S1, one of which involves formation of a peroxide intermediate (the OOH dissociation pathway or the O_2_ associative 4e^−^ pathway), and one that proceeds directly to OH without peroxide formation (the O_2_ direct dissociation pathway or the O_2_ dissociative 4e^−^ pathway). As shown in Figure S2 and listed in Table S1, the activation energy barrier for the O_2_ direct dissociation pathway is 0.715 eV, the activation energy barrier for the OOH dissociation pathway is 0.821 eV and 0.348 eV, respectively. This suggests that the OOH dissociation pathway is not possible for the four-electron pathways of ORR reaction on the pIh Ag_32_Cu_6_ cluster. Hence, in this work only the O_2_ direct dissociation is considered.

The adsorption of an oxygen molecule on the pIh Ag_32_Cu_6_ core-shell nanoparticle must be considered for identification of the O_2_ dissociation catalytic properties in ORR. Therefore, we have considered four typical nonequivalent positions for the O_2_ adsorption, which are named as following: Bridge 1 (B1), B2, B3 and B4 sites. The bridge position refers to an O_2_ molecule situated in the middle of Ag-Ag dimer. Similarly, hollow1 (H1), H2 and H3 sites represent one O atom situating on the top of (111) facet.

**Figure 4:**
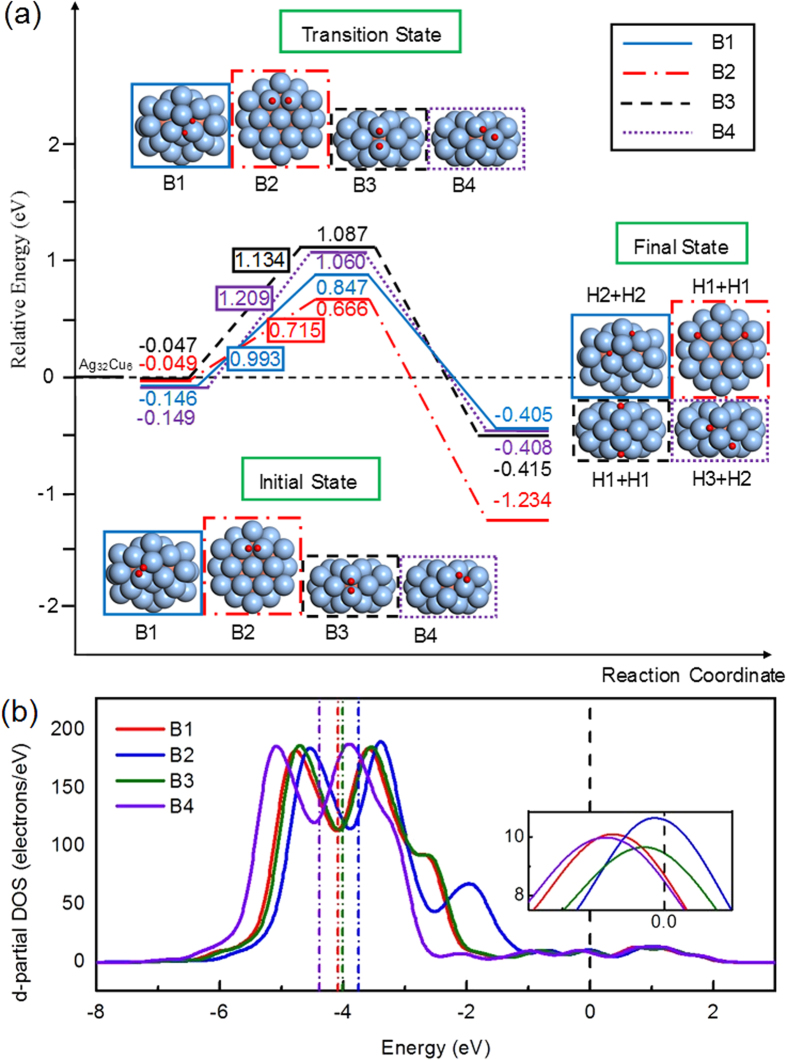
(**a**) Profiles of potential-energy surfaces of four oxygen dissociation pathways on the pIh Ag_32_Cu_6_ core-shell cluster. (**b**) Center of *d*-band and density of states at the Fermi level (inset) of four O_2_-cluster adsorption configurations.

To investigate the oxygen dissociation reaction of ORR, four O_2_ dissociation energy paths for B1-4 sites have been calculated. Figure 4(a) shows the calculated dissociation potential-energy surfaces and the binding energy levels of the initial, transition and final states. Table 1 tabulates the oxygen dissociation reaction parameters. Among these four adsoption configurations, we notice that the adsorption energy on B4 site has a highest value of −0.149 eV, and also the highest value of 1.209 eV for the dissociation barrier, and an exothermicity of 0.259 eV, dissociating to H2 and H3 sites. The B1 site, which has similar adsorption energy to B4 site, −0.146 eV, dissociates to two H2 sites with barrier of 0.993 eV and exothermicity of 0.259 eV. The O_2_ on B2 and B3 sites with smaller adsorption energies is bond-cleavage from two bridge sites to two hollow sites with barriers of 0.715 and 1.134 eV, and exothermicities of 1.088 and 0.368 eV, respectively. It is clear that the most favorable pathway for the O_2_ dissociation is B2 site with an activation energy barrier of 0.715 eV.

The interaction strength of atoms and molecules with metal surface to a large extent is controlled by the location of the *d*-band center. In order to elucidate further that B2 site is optimal to display a good catalytic behavior, we address the electronic structure of these four adsorption configurations and calculate the position of the *d*-band center relative to the Fermi energy for these different sites, shown in Fig. 4(b) and listed in Table 1. The *d*-band center of B2 site is −3.395 eV, which is closest to the Fermi energy. According to the Hammer-Nørskov *d*-band model^19^,^20^, the B2 site is predicted to have a higher chemical activity.

Frontier orbital theory^21^ establishes that the states of a metal which is directly involved in electron transfer with the adsorbate are closest to the Fermi energy. Therefore, the density of states at the Fermi energy level gives rise to indicators of the chemical activity. We notice that the density of states at the Fermi energy level is maximal for B2 site. For this reason, the highest of the catalytic activity observed for B_2_ site is attributed to a maximal charge transfer, which is agreeable with the Mulliken charge of O_2_ at B_2_ site of the cluster, as listed in Table 1. It can be inferred that the pIh Ag_32_Cu_6_ cluster is able to activate O_2_ by charge transfer.

Based on above results, the pIh Ag_32_Cu_6_ core-shell cluster may be a good candidate for ORR catalysts because it has optimal oxygen adsorption energy of −0.049 eV and a low activation energies of 0.715 eV. For the similar oxygen dissociation pathway from B to H site on the TO Ag_32_Cu_6_ core-shell cluster, it is reported that the adsorption energy is −0.093 and the activation energy is 0.926 eV^12^. Such a high activation energy of Ag_32_Cu_6_ core-shell cluster may be due to the fact that TO structure is not a global minimum for 38 atom Ag-Cu cluster, so it is not solid to say that Ag_32_Cu_6_ core-shell nanoparticles are not suitable as ORR catalysts because all related results are calculated from TO structure.

Comparing the activation energy of oxygen dissociation of the Ag_32_Cu_6_ core-shell cluster with those of Pt_32_Ti_6_ nanoparticles^9^, Ag_32_Cu_6_ and Pt_32_Ti_6_ nanoparticles have similar activation energy of oxygen dissociation (0.715 eV and 0.62 eV, respectively). In the case of the adsorption energy, pIh-Ag_32_Cu_6_ nanoparticles have a much lower adsorption energy (−0.049 eV) than that of Pt_32_Ti_6_ nanoparticles (−1.76 eV). However, as listed in Table S2, for the pIh Ag_32_Cu_6_ core-shell cluster, the adsorption energy of O_2_ (−0.049 eV) is small but can be enhanced with the net negative charge from the cathode up to −0.602. Moreover, the dissociation barrier on the B2 site of the pIh Ag_32_Cu_6_ cluster is not changed much with net charge ranging 0.715–0.724 eV, suggesting that the Ag-Cu clusters deposited on inert supports would efficiently activate O_2_ for a cathode catalyst in alkaline fuel cells.

Due to the fact that the adsorption energy of O_2_ molecule on clusters is related to outside conditions, such as net charge transfer, temperature variation and oxygen content of atmosphere, strong adsorption of O_2_ molecule on clusters is not a sufficient condition for O_2_ dissociation. In case of the lower adsorption energy on pIh-Ag_32_Cu_6_ nanoparticles, a fraction of adsorbed molecules will dissociate and another fraction will desorb. However, as shown in Table S1 and Fig. 4(b), the adsorption energy of O_2_ molecule on clusters can be easily controlled by the net charge from the cathode and the oxygen content in the atmosphere, while the interaction strength of atoms and molecules with metal surface is tuned by the location of the *d*-band center, which implies that the adsorption energy can be intrinsically enhanced by the strain effect and electronic effect in the catalyst. This suggests that Ag_32_Cu_6_ nanoparticles may be a good catalyst for the ORR. Therefore, it can be theoretically confirmed that Ag-Cu nanoalloy is a potential candidate to substitute noble Pt-based catalyst in alkaline fuel cells.

### ORR activity of Ag-Cu nanoalloys

**Figure 5:**
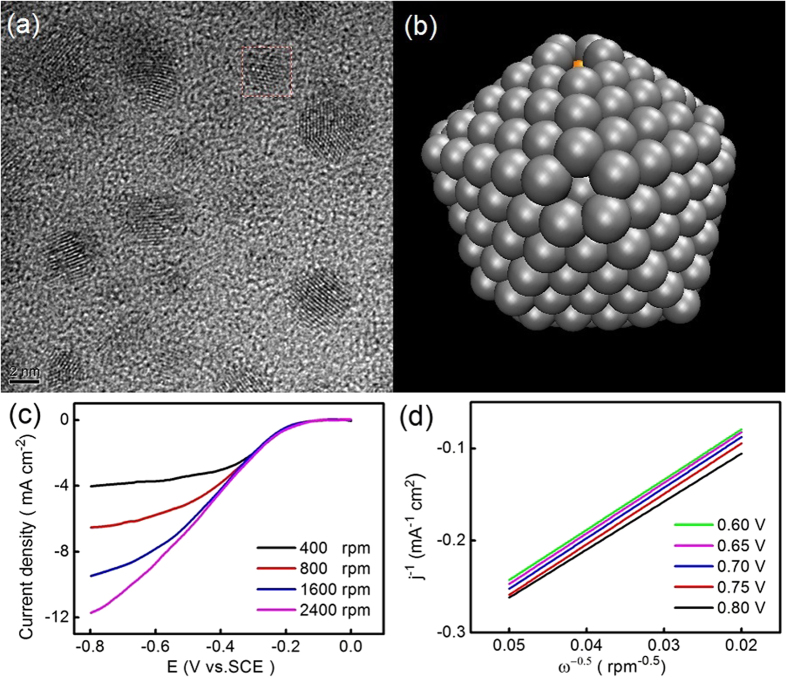
(**a**) HRTEM image of Ag-Cu nanoalloy via PLD, (**b**) Atomic model for 561-atom Ag-Cu cluster, (**c**) ORR polarization curves for Ag-Cu nanoalloy (**d**) Koutecky Levich plots collected from the ORR for Ag-Cu nanoalloy.

To verify the theoretical prediction above, we synthesis the Ag-Cu nanoalloy in a home-made pulse laser deposition equipment. Figure 5(a) shows that these nanoparticles are 1–5 nm in diameter with an average size of 2.58 nm in the amorphous films. Figure S3(a) shows a typical Ag-Cu nanoparticle with size of 2.6 nm outlined by a red rectangle, its Fast Fourier Transform (FFT) image in the Figure S3(b) clearly illustrates the characteristic of core-shell structure, as indicated by two set of aligned diffraction spots corresponding to Cu and Ag phase with cube on cube epitaxial relationship. Moreover, the inverse of Fast Fourier Transform (IFFT) image in the Figure S3(c) clearly shows the location of Ag and Cu fringes is Cu core and Ag shell. As shown in Figure S4, the Ag-Cu core-shell cluster possesses an atomic ratio of Ag to Cu is 80:20.

In agreement with the shape, size and atomic ratio, a Ag_444_Cu_147_ nanoparticle with N = 561 atoms and 2.6 nm diameter can be generated by GA method like the Ag_32_Cu_6_ cluster. As shown in Figure S5, the Ag_444_Cu_147_ nanoparticle has a pure Cu core with a size of 147 atoms and two layers of Ag shell at the subsurface and surface. The Ag-Cu nanoparticle formed during the PLD experiment is a rapidly quenched Ag-Cu alloy and not a perfect core-shell Ag_444_Cu_147_ cluster. In order to examine their atomic-level structures, MD simulations were performed. For each quench experiment, the starting liquid state is obtained by melting the Ag_444_Cu_147_ cluster at a slow rate of 0.8 K/ns and then rapidly quenched at 800 K/ns, Figure S6 shows the evolution of total energy during the quenching of the Ag_444_Cu_147_ nanoparticle.

Figure 5(b) shows the atomic structure of the quenched Ag_444_Cu_147_ nanoparticle, which has the 147-atom Cu core off-centered in the nanoparticle and a large portion of single Ag atom layer on the nanoparticle surface (for more information refer to Figure S7 and Table S3). The size of 2.6 nm in Ag_444_Cu_147_ cluster is the best size for ORR activity of Pt cluster^22^, and the single Ag atom layer on the surface of Ag_444_Cu_147_ nanoparticle is similar to the Ag_32_Cu_6_ cluster, which has a complete single Ag atom layer on the core-shell structure.

Figure 5(c) shows the ORR performance for the Ag_444_Cu_147_ catalysts at different rotating rates. The rotating disk electrode (RDE) data shows that the current density increases with increasing rotation rate, indicating that the limiting current density is controlled by the diffusion distance of oxygen to the Ag-Cu catalyst surface. The number (n) of electrons transferred on the Ag_444_Cu_147_ catalyst during ORR, which determines the catalytic efficiency, is calculated by the Koutechy–Levich plots in Fig. 5(d). The result with n = 3.9 indicates that the ORR catalysed by the Ag_444_Cu_147_ catalyst occurs through a four-electron pathway, which can be expressed as following:


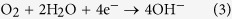


The four-electron pathway of the ORR process in alkaline electrolytes indicates that O_2_ molecule is directly reduced into OH^−^ ions through O_2_ bond cleavage on the Ag_444_Cu_147_ nanoalloy, which corresponds to the catalytic pathway predicted by the present DFT calculation, and the O-O bond breaking on the polyicosahedral (pIh) Ag_32_Cu_6_ core-shell cluster.

## Conclusion

We have shown that the GA described here is both efficient and reliable for finding the global minima of Ag-Cu nanoclusters with 38 atoms geometries, and the global optimization structure of Ag_32_Cu_6_ is the polyicosahedral (pIh) core-shell cluster where a isomer for Ag_32_Cu_6_ is the truncated octahedron (TO) core-shell cluster. Previously, only the TO Ag_32_Cu_6_ core-shell cluster is calculated for its ORR activity. In order to test the structural stability for the pIh and TO Ag_32_Cu_6_ core-shell cluster, DFT calculations and molecular dynamics simulations have been performed. It is shown that the TO cluster is a metastable isomer, and its transition from order to disorder is thermodynamically favorable.

We have also analyzed the O_2_ dissociation processes on the pIh Ag_32_Cu_6_ nanoclusters. The O_2_ bond cleavage might be favored on B2 sites having activation energy barrier of 0.715 eV. The *d*-band center of B2 site is closest to the Fermi level in all sites, and the density of states (DOS) at Fermi level of B2 site is the highest one. Thus, the Ag_32_Cu_6_ pIh core-shell cluster is a promising candidate for an ORR catalyst.

## Method

### Gupta potential model

The atomistic potential employed in our calculations is derived from the second-moment approximation to the tightbinding (SMATB) model^23^,^24^, which is often denoted as the Gupta potential^25^ in the literature. The SMATB potential is many-body potential, because it cannot be written as the sum of pair terms. Within the SMATB model, the contribution to the total potential energy of atom is made up of a many-body (nonlinear) bonding term and a repulsive Born-Mayer pair term. Its analytical form is defined as follows:


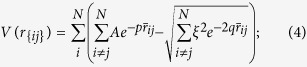


where *N* is the number of atoms, 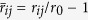, and r_*ij*_ is the distance between the atoms at sites *i* and *j*. The Gupta potential parameters *A*, *ξ*, *p*, *q* used in this study are listed in Table 2^26^. The homo-atomic interactions were fitted to several bulk experimental values. For hetero-atomic interactions, *A* and *ξ* are fitted to the solubility energy, and *p* and *q* are taken as averages of the values of the pure constituents. It should be noted that the interaction potential has been used without a distance cutoff, though the potential was originally fitted^26^ by imposing a cutoff between the second-neighbor Ag distance of Ag and the third-neighbor Cu distance. Eliminating the cutoff may change isomer ordering in the cases where energy differences fall below 0.1 eV, but for 38-atom clusters we do not expect this to affect the results qualitatively.

### The genetic algorithm for global optimization

The global optimization searches are performed using the genetic algorithm (GA), which have been described in detail previously^27^,^28^,^29^. GA is an optimization technique based on the principles of natural evolution. It is inspired by the natural selection process in a competitive survival environment. The GA belongs to the class of evolutionary algorithms, which also includes evolution strategies, differential evolution and genetic programming.

**Figure 6:**
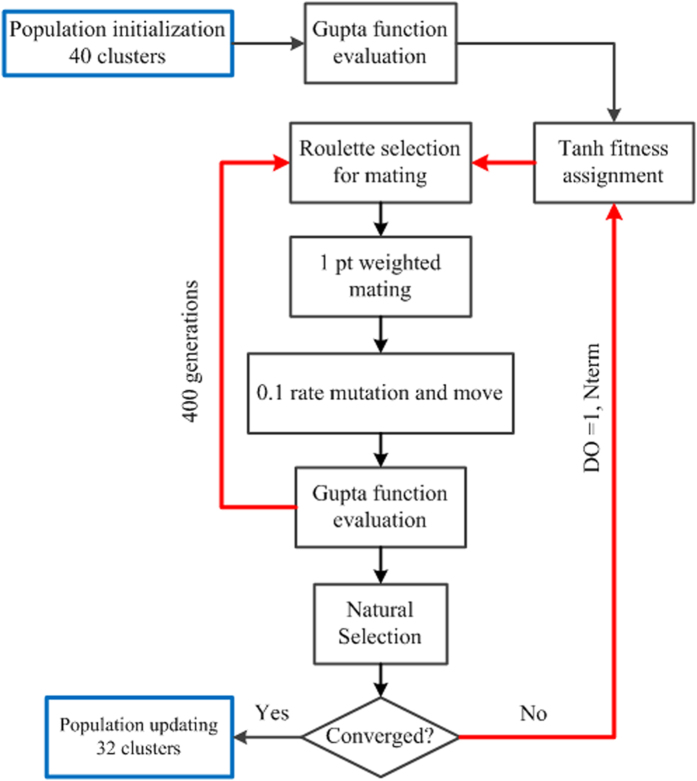
Schematic flow chart for the cluster geometry optimisation genetic algorithm (GA) program.

To represent the operation of our cluster geometry optimization GA program, a flow chart is shown in Fig. 6. The cluster GA operates as follows:

1. For a given cluster nuclearity, 40 clusters are generated randomly to form the initial population, population size = 40 clusters.
2. The Gupta energy function is defined, along with the crossover, mutation schemes and the GA parameters. Each cluster is then relaxed by locally minimizing the cluster potential energy.
3. Each cluster is assigned to a fitness value using the tanh function of its total potential energy and sorted according to its fitness. The low-energy structures have high fitness, fitness function = tanh fitness relationship.
4. For each generation,
  - Parents are chosen with a roulette wheel method depending on their fitness;
  - Offspring are generated from the selected parents using 1 point weighted scheme, that is, the parent clusters are cut on an atomic position based on the fitness value, crossover (mating) rate = 0.8, crossover type = one-point weighted;
  - Mutation is carried out on the set of offspring with a move operation, the number of mating is generally set to 10% of the population size, 1/3 atoms of the cluster to be moved to new random position, mutation rate = 0.1, mutation type = mutation move;
  - The population is ranked according to fitness;
  - Some of the old individuals are replaced by new individuals, depending on their relative fitness.
5. This process is repeated until a convergence criterion is reached or a maximum number of 400 generations is reached.
6. The individuals in the last generation are stored.

During the geometry optimization, 20 GA runs are carried out for each composition with different random number seeds and the resultant structures are then analyzed by comparing the average binding energy, *E*_b_, for an N-atom cluster, which is defined as:


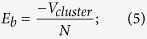


where *V*_cluster_ is the total cluster potential energy.

### Molecular dynamics simulations

A constant-energy molecular dynamics simulations (MD) is used to study the thermal stability of Ag-Cu nanoalloy. The energetic model in MD simulation is the Gupta potential with parameters listed in Table 2. Using the same potential model allows us a seamless study of static structures by GA and dynamics behavior by MD on long time scales. The Newton equation is solved by the velocity Verlet algorithm. Initial drift velocity of atoms are sampled according to the Maxwell distribution. The translational and rotational motion is eliminated. The temperature is increased by scaling up to the velocities in a steplike manner. The time step of the MD run is taken as 7 fs. During the computational scheme, the different modes of thermal stability are directly observed on the atomic configuration snapshots and the physical properties are tracked by the caloric curves calculated by taking time averages, <*E*_t_> during a single MD run of the total energy *E*.

### Density functional theory (DFT) calculations

DFT local relaxation calculations are performed by the Dmol3 package included in the software Materials Studio^30^. The geometric structures of all clusters obtained by global optimization are optimized by using the generalized-gradient approximation (GGA) exchange-correlation functional is provided by revised Perdew-Burke-Ernzerhof (RPBE) in DFT level^31^. The GGA functional has been checked to reproduce surface energies in good agreement with experiments for both Ag and Cu. This functional describes atomic or molecular binding to transition metal well and is widely used^32^,^33^,^34^,^35^. The Kohn-Sham equation is expanded in a double numeric quality basis set with polarization functions (DNP)^31^. The orbital cutoff range is 5.0 Å, and a Fermi smearing of 0.001 Ha (1Ha = 27.212 eV). The DFT Semi-core Pseudo-potential is used to treat the electrons of heavy Ag and Cu. In order to obtain well-converged geometric structures, the SCF (Self-Consistent Field) convergence within 10^−5^, the energy, maximum force and maximum displacement convergence criterion are set to 10^−5^ Ha, 0.002 Ha/ Å and 0.005 Å, respectively. The dissociation energy paths for ORR are obtained by LST/QST tools.

### Experimental preparation and characterization

The Ag–Cu nanoalloy is synthsised on nickel foam by pulse laser deposition. All nickel foams are pre-processed by acetone (3 hours), 10% H_2_SO_4_ (15 minimum), distilled water (10 minimum) and vacuum drying (2 hours). The targets are irradiated by a nanosecond Q-switched Nd:YAG laser beam (EKSPLA, Lithuania) with a wavelength of 266 nm and a pulse duration of 3–6 ns. The laser beam diameter is about 1 mm with an energy density about 200 mJ/pulse. Both the target and substrate are rotated at the speed of 5 rpm during deposition, and the target is irradiated for 2 minutes at 10 Hz to clear away the oxide on the metal prior to each growth run, and then is irradiated at a 200 mJ/pulse to deposit the Ag–Cu nanoalloy onto the nickel foam. In all cases, the substrate-to-target distance is fixed at 5 cm. Each Ag-Cu catalyst is deposited with 36000 laser pulse, the deposition rate of Ag-Cu catalyts is precalibrated by a quartz crystal monitor. The structural features of the synthesized catalysts are examined by transmission electron microscopy (TEM, FEI Tecnai F30 G^2^) at 300 kV.

### Electrochemical measurements

The electrocatalytic activities of the catalysts are studied at room temperature by rotating disk electrode (RDE) polarization curves. The experiments are performed with a classic three-electrode cell containing a saturated calomel electrode (SCE) as reference electrode, a Pt counter electrode and the Ag-Cu nanoalloys supported on Ni foam as the working electrode in the CHI660C electrochemical workstation. The electrolyte is 0.1 mol L^−1^ KOH aqueous solution. The experiments are performed over the potential range of 0 to −0.8 V at a scanning rate of 10 mV s^−1^ and the rotation rates are monitored at 400, 800, 1600 and 2400 rpm.

## Additional Information

**How to cite this article**: Zhang, N. *et al.* Global optimization and oxygen dissociation on polyicosahedral Ag_32_Cu_6_ core-shell cluster for alkaline fuel cells. *Sci. Rep.* **5**, 11984; doi: 10.1038/srep11984 (2015).

## Supplementary Material

Supplementary Information

## Figures and Tables

**Table 1:** Calculated activation energy (*E*_*act*_) and reaction energy (*E*_*rea *_) for typical reaction pathways of O_2_ bond cleavage on the pIh Ag _32_Cu_6_ core-shell cluster, and Mulliken charge (Δ *q*) of O_2_ on cluster, including center of the *d*-band relative to the Fermi energy and density of states (DOS) at the Femi energy for the pIh Ag_32 _Cu_6_ core-shell cluster under four O_2_-cluster adsorption configurations (B1-B4).

Reaction pathway	*E*_*act*_ /eV	*E*_*rea*_ /eV	Δ*q*/|e|	Center of *d*-band /eV	DOS at Fermi energy /eV^−1^
B1→H2+H2	0.993	−0.259	−0.349	−4.346	8.679
B2→H1+H1	0.715	−1.088	−0.407	−3.689	10.583
B3→H1+H1	1.134	−0.368	−0.283	−3.922	9.429
B4→H3+H2	1.209	−0.259	−0.274	−4.053	8.459

**Table 2:** Gupta potential parameters used in this study^a^.

Parameters	Cu-Cu	Ag-Ag	Cu-Ag
*A* (eV)	0.0894	0.1031	0.0980
*ξ* (eV)	1.2799	1.1895	1.2274
p	10.55	10.85	10.70
*q*	2.43	3.18	2.8050
*r*0 (Å)	2.556	2.8921	2.72405

^a^Ref. 26.
