# Supplementary material for: Global optimization and oxygen dissociation on polyicosahedral Ag32Cu6 core-shell cluster for alkaline fuel cells
Source: Sci Rep. 2015 Jul 7;5:11984. doi: 10.1038/srep11984 (PMC4493688; doi:10.1038/srep11984)
Supplement: Supplementary Information [file srep11984-s1.doc]

Supporting Information

**Global optimization and oxygen dissociation on polyicosahedral Ag_32_Cu_6_ core-shell cluster for alkaline fuel cells**

N. Zhang, F. Y. Chen* & X.Q. Wu

State Key Laboratory of Solidification Processing, Northwestern Polytechnic University, Xian 710072, China,

*Correspondence and requests for materials should be addressed to F. Y. Chen, Telephone/Fax: +29 88492052,fuyichen@nwpu.edu.cn.

**Contents**

Figure S1 Low energy structure for Ag_30_Cu_8_ searched by GA.

Scheme S1 Possible four-electron pathways of ORR reaction in alkaline solution.

Figure S2 The 3th and 4th step of the series pathway for four-electron ORR reaction.

Figure S3 TEM images of Ag-Cu nanoparticles: (a) HRTEM, (b)FFT and (c) IFFT corresponding to the nanoparticle marked in red rectangle.

Figure S4 STEM-HAADF line-scanning of Ag-Cu NPs

Figure S5 Structure model of a perfect core-shell Ag_444_Cu_147_ nanoparticle from GA.

Figure S6 MD heating and cooling curves for a perfect core-shell Ag_444_Cu_147_ to model the PLD process.

Figure S7 Structure models of the quenched Ag_444_Cu_147_ core-shell nanoparticles.

Table S1 Direct O_2_ and OOH dissociation step for O_2_ activation on pIh cluster

Table S2 O_2_ activation on the position B2 of pIh Ag_32_Cu_6_ with charge transfer of 0, -1 and -2

Table S3 Atomic coordination of Ag_444_Cu_147_ nanoalloy


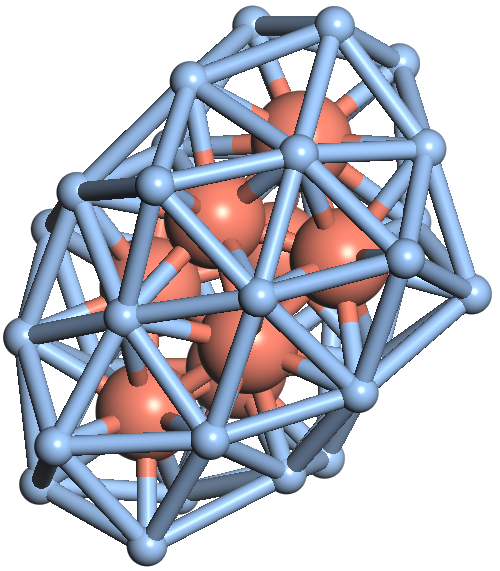

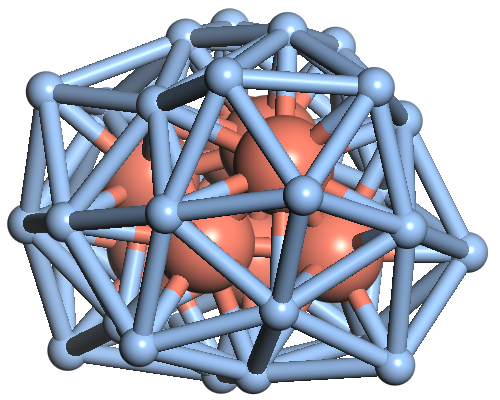


Figure S1 Low energy structure for Ag_30_Cu_8_ searched by GA.

O_2_(g)

O_2_(ad)

O(ad)

H_2_O

OH(ad)

OH^-^

②

③

④

⑤

H_2_O

H_2_O

OOH(ad)

⑥

①

Scheme S1 Possible four-electron pathways of ORR reaction in alkaline solution.


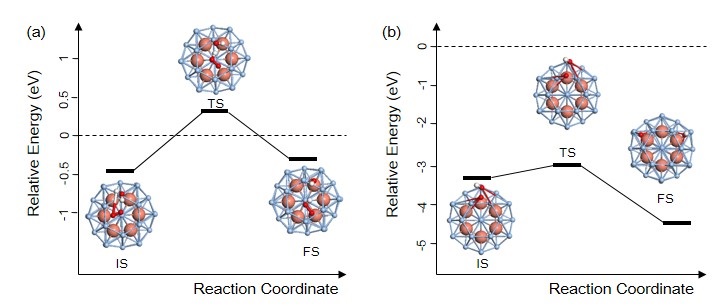


Figure S2 The 3th and 4th step of the series pathway for four-electron ORR reaction.


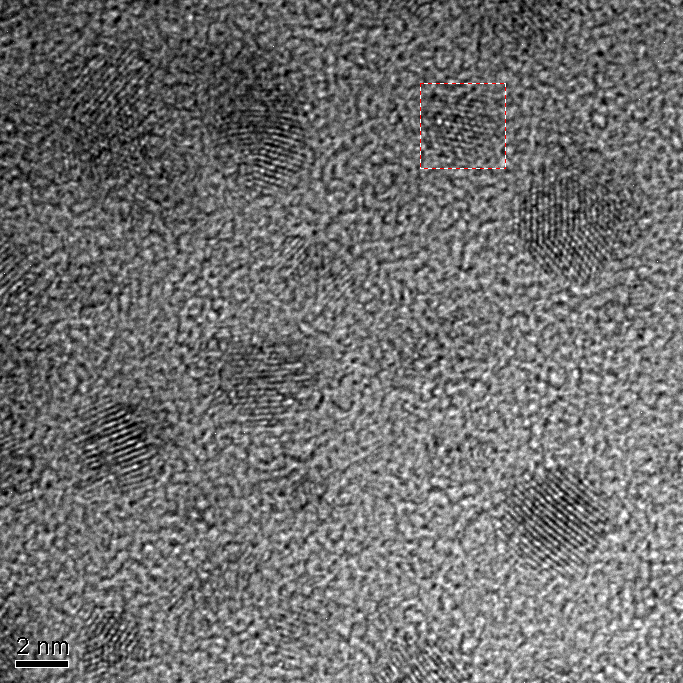


(a)


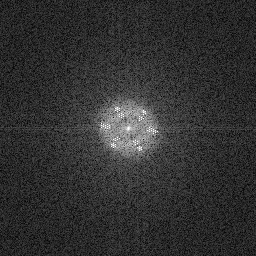


(b)


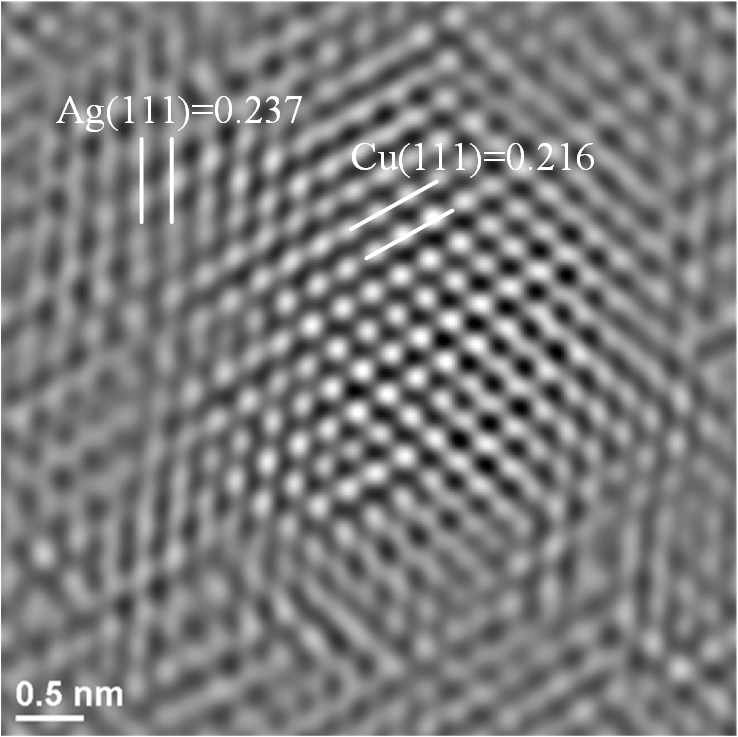


(c)

Figure S3 TEM images of Ag-Cu nanoparticles: (a) HRTEM, (b)FFT and (c) IFFT corresponding to the nanoparticle marked in red rectangle.


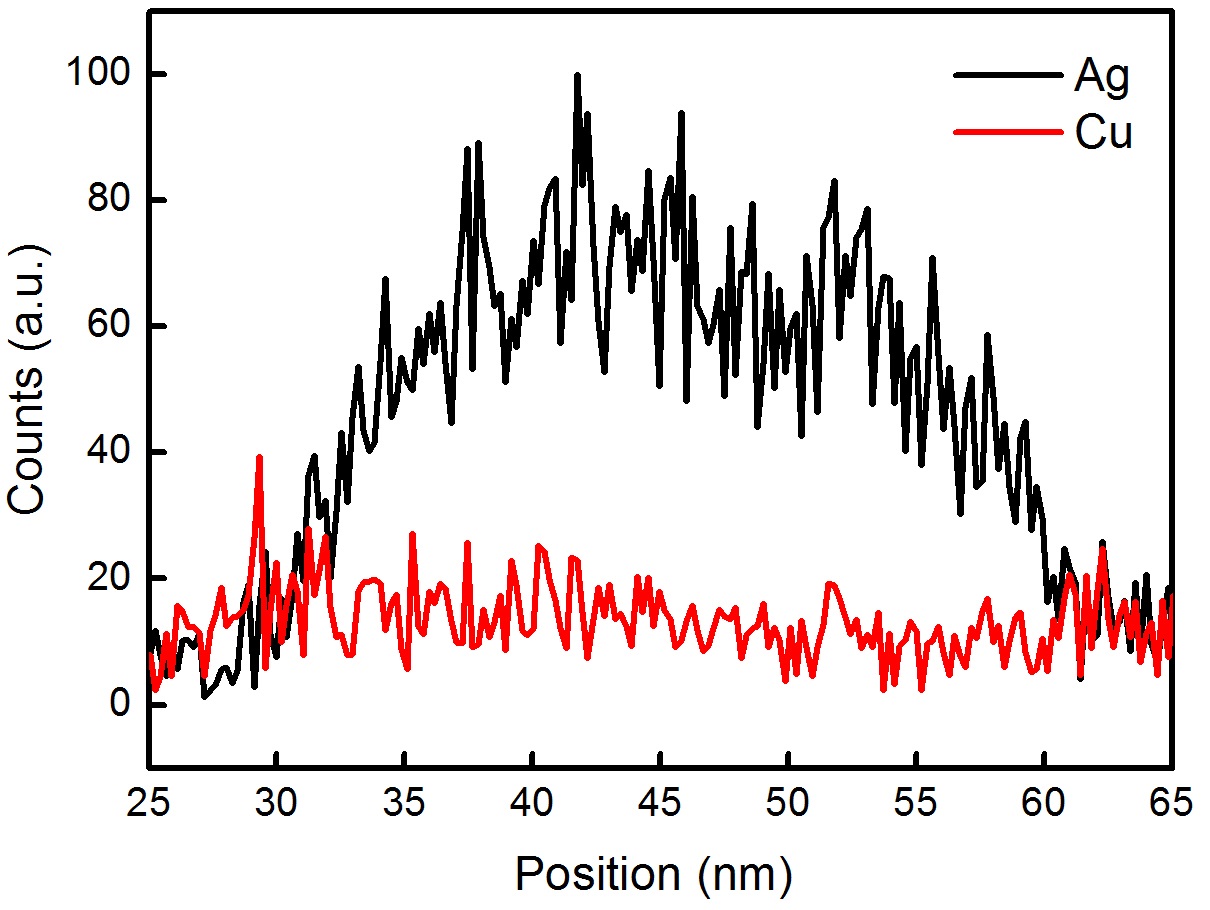


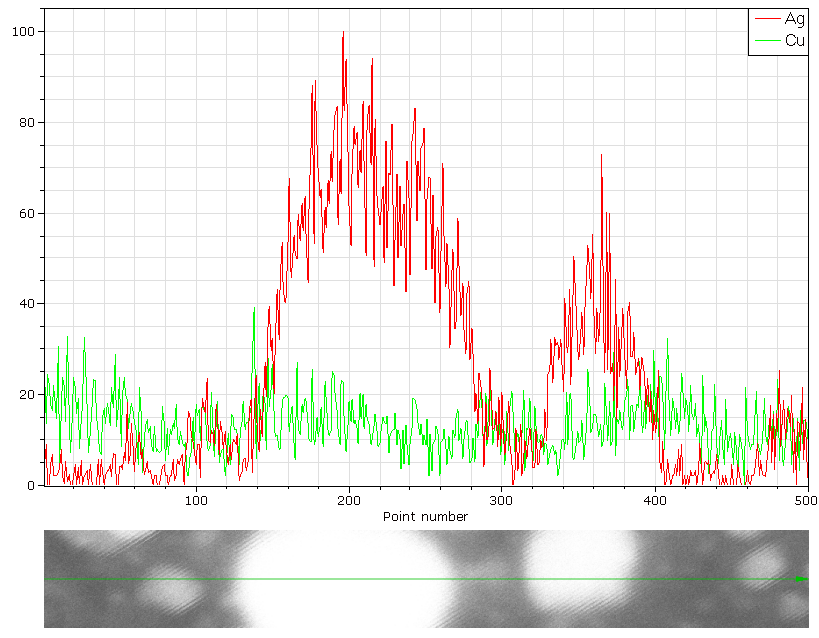


Figure S4. STEM-HAADF line-scanning of Ag-Cu NPs


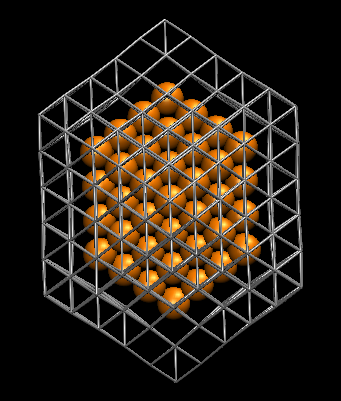

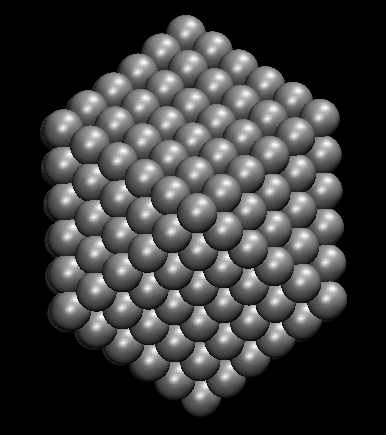


Figure S5 Structure model of a perfect core-shell Ag_444_Cu_147_ nanoparticle from GA.

Figure S6 MD heating and cooling curves for a perfect core-shell Ag_444_Cu_147_ to model the PLD process.


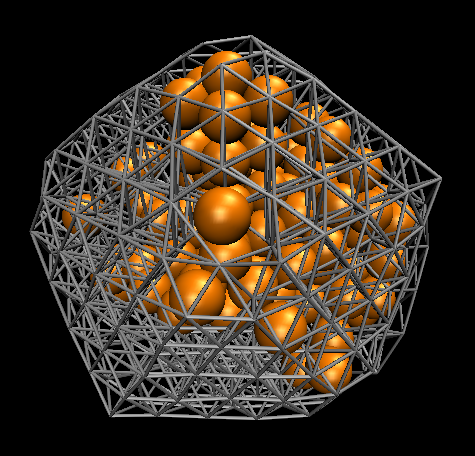

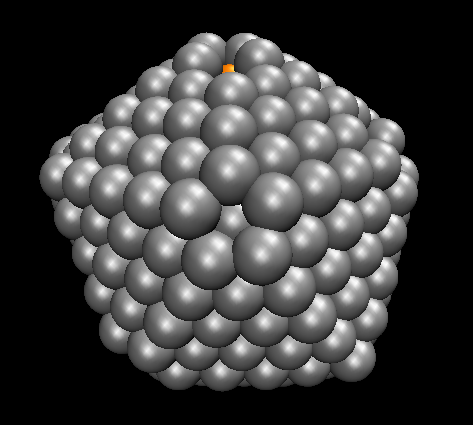


Figure S7 Structure models of the quenched Ag_444_Cu_147_ core-shell nanoparticles.

Table S1 Direct O_2_ and OOH dissociation step for O_2_ activation on pIh cluster

| O_2_ dissociation path | *E_ads_* (eV) | *E_act_* (eV) | *E_rea_* (eV) |
| --- | --- | --- | --- |
| Direct O_2_ dissociation (step 2): O_2_ →2O | -0.049 | 0.715 | -1.088 |
| OOH dissociation (step 3): O_2_+H_2_O→OOH | -0.493 | 0.821 | 0.181 |
| OOH dissociation (step 4): OOH→O+OH | -3.365 | 0.348 | -1.161 |

Table S2 O_2_ activation on the position B2 of pIh Ag_32_Cu_6_ with charge transfer of 0, -1 and -2

| Net charge | *E_ads_* (eV) | *E_act_* | *E_rea_* |
| --- | --- | --- | --- |
| 0 | -0.049 | 0.715 | -1.088 |
| -1 | -0.385 | 0.719 | -1.093 |
| -2 | -0.602 | 0.724 | -1.011 |

Table S3 Atomic coordination of Ag_444_Cu_147_ nanoalloy

| Cu | -2.15949 | 35.08017 | -7.73735 |
| --- | --- | --- | --- |
| Cu | 2.79869 | 31.64864 | -9.52690 |
| Cu | -2.75775 | 36.00838 | -15.38753 |
| Cu | 1.47233 | 30.72149 | -12.55863 |
| Cu | -6.56056 | 30.92901 | -4.19238 |
| Cu | -2.34024 | 29.92463 | -4.25615 |
| Cu | -2.39409 | 28.06995 | -8.25114 |
| Cu | -0.41873 | 29.07614 | -5.64849 |
| Cu | -4.82403 | 35.81099 | -14.29228 |
| Cu | -2.82818 | 37.28160 | -9.00783 |
| Cu | -2.67728 | 31.33580 | -12.13648 |
| Cu | -1.63056 | 30.33248 | -1.78399 |
| Cu | -10.24489 | 36.59582 | -7.74701 |
| Cu | -9.38255 | 35.09668 | -3.60841 |
| Cu | -0.10873 | 31.31982 | -3.83547 |
| Cu | -5.04108 | 39.73077 | -9.82074 |
| Cu | -3.18610 | 35.82352 | -12.34411 |
| Cu | -1.75158 | 32.52091 | -10.18370 |
| Cu | -14.22060 | 29.10777 | -15.86717 |
| Cu | -2.07891 | 32.24404 | -5.07854 |
| Cu | -7.15784 | 35.45616 | -18.48510 |
| Cu | -9.20532 | 34.52513 | -8.59389 |
| Cu | -14.14735 | 34.53184 | -5.57200 |
| Cu | -1.52125 | 25.58276 | -9.36102 |
| Cu | -4.07914 | 37.80599 | -11.11088 |
| Cu | -8.94968 | 36.31898 | -10.22391 |
| Cu | -8.38810 | 36.98475 | -6.09310 |
| Cu | -11.59110 | 34.60931 | -7.20671 |
| Cu | -2.07406 | 35.09531 | -10.29996 |
| Cu | -5.27913 | 37.50594 | -8.67437 |
| Cu | -12.72621 | 33.16388 | -9.08966 |
| Cu | -11.46688 | 30.81764 | -9.75763 |
| Cu | -2.49227 | 39.63463 | -10.22465 |
| Cu | -2.56458 | 35.36598 | -2.23421 |
| Cu | -2.55495 | 29.12392 | -11.24497 |
| Cu | 0.69156 | 32.50687 | -11.07817 |
| Cu | -2.33111 | 32.61551 | -2.42491 |
| Cu | -2.20148 | 30.40589 | -9.09964 |
| Cu | -8.41860 |  |  |
| Cu | -13.83479 | 30.76072 | -8.30228 |
| Cu | -1.50819 | 37.38157 | -11.39143 |
| Cu | -11.27638 | 37.43580 | -11.31055 |
| Cu | -4.10734 | 31.40644 | -10.04588 |
| Cu | -8.01750 | 32.64495 | -9.50809 |
| Cu | -3.44369 | 37.11229 | -6.45953 |
| Cu | -1.83118 | 32.50137 | -7.73487 |
| Cu | -12.94782 | 32.52327 | -6.55621 |
| Cu | -8.50134 | 32.80060 | -4.46961 |
| Cu | -4.24701 | 31.16759 | -2.97745 |
| Cu | -5.82821 | 33.12077 | -8.14747 |
| Cu | -4.43823 | 35.62999 | -10.05302 |
| Cu | -5.01306 | 35.47673 | -2.71416 |
| Cu | -9.31998 | 32.90829 | -13.10555 |
| Cu | -3.61237 | 33.63545 | -11.40777 |
| Cu | -3.99868 | 33.33642 | -3.98201 |
| Cu | -5.98892 | 37.08807 | -6.47486 |
| Cu | -0.68064 | 31.49968 | -13.28893 |
| Cu | -6.31105 | 33.34389 | -3.15724 |
| Cu | -7.39787 | 33.39311 | -16.75194 |
| Cu | -1.01865 | 35.89766 | -13.38776 |
| Cu | -6.09613 | 37.30235 | -3.96065 |
| Cu | -7.80638 | 37.31135 | -8.38458 |
| Cu | 1.48822 | 29.50630 | -9.92334 |
| Cu | -10.70491 | 32.81082 | -2.85631 |
| Cu | -2.48394 | 27.73971 | -5.83000 |
| Cu | -7.44179 | 39.59434 | -9.42256 |
| Cu | 0.96126 | 33.74093 | -13.03272 |
| Cu | -12.41234 | 27.50666 | -17.43546 |
| Cu | -15.28946 | 32.49401 | -5.24698 |
| Cu | -0.46117 | 30.42161 | -11.01117 |
| Cu | -10.54971 | 32.59090 | -8.07068 |
| Cu | -6.66278 | 39.84540 | -11.86441 |
| Cu | -0.09075 | 34.03706 | -6.36256 |
| Cu | -7.10033 | 25.75582 | -6.89627 |
| Cu | 3.09703 | 32.62042 | -12.01253 |
| Cu | -6.72465 | 35.90108 | -15.91282 |
| Cu | -4.38364 | 31.03976 | -5.35716 |
| Cu | -11.77970 | 34.48758 | -4.62430 |
| Cu | -3.98215 | 24.53545 | -9.04104 |
| Cu | 1.74896 | 32.75940 | -4.96323 |
| Cu | -2.53789 | 25.49430 | -6.99716 |
| Cu | -12.73021 | 36.54004 | -6.18760 |
| Cu | -0.37516 | 26.86689 | -7.36997 |
| Cu | -5.74953 | 35.43920 | -12.04771 |
| Cu | -0.60491 | 29.02286 | -12.94791 |
| Cu | -3.04032 | 23.22089 | -5.64273 |
| Cu | -3.51850 | 37.15141 | -3.82008 |
| Cu | -0.02120 | 31.49231 | -6.40623 |
| Cu | -9.18579 | 28.17981 | -3.99603 |
| Cu | -11.23360 | 35.05322 | -1.99421 |
| Cu | -4.88816 | 35.40326 | -5.11157 |
| Cu | -0.56585 | 27.93259 | -10.22960 |
| Cu | -7.11688 | 35.11558 | -4.59360 |
| Cu | -7.95748 | 34.40661 | -11.35748 |
| Cu | -2.14644 | 30.18000 | -6.72282 |
| Cu | -4.59709 | 26.73527 | -7.83966 |
| Cu | -1.32185 | 33.76373 | -12.26587 |
| Cu | -10.52667 | 36.59503 | -5.27332 |
| Cu | -4.06466 | 31.47300 | -7.68861 |
| Cu | -3.63265 | 33.55434 | -9.00399 |
| Cu | -10.27029 | 33.31434 | -10.55971 |
| Cu | -2.95128 | 29.89076 | -13.89216 |
| Cu | -4.37958 | 29.09667 | -9.21061 |
| Cu | -0.13017 | 33.93125 | -9.05603 |
| Cu | 1.51627 | 27.98355 | -11.90232 |
| Cu | 2.62047 | 27.88737 | -4.65988 |
| Cu | -3.14198 | 34.05807 | -14.02291 |
| Cu | -6.84934 | 35.29934 | -9.45953 |
| Cu | -12.31096 | 30.83810 | -15.18849 |
| Cu | 1.79804 | 32.93611 | -7.59292 |
| Cu | -4.42606 | 28.84845 | -4.37365 |
| Cu | -6.08384 | 32.92998 | -5.58655 |
| Cu | -9.53688 | 34.44575 | -6.00843 |
| Cu | -8.36559 | 32.62126 | -7.02889 |
| Cu | -0.25374 | 29.24615 | -8.10572 |
| Cu | -6.60456 | 37.39405 | -10.72377 |
| Cu | 3.91760 | 28.89288 | -6.45408 |
| Cu | 1.98246 | 30.34868 | -7.63938 |
| Cu | -11.45930 | 35.47680 | -9.70924 |
| Cu | -4.73388 | 38.90709 | -5.16612 |
| Cu | 0.69074 | 26.62400 | -13.95439 |
| Cu | -3.86167 | 33.51936 | -6.47141 |
| Cu | -10.69315 | 32.36721 | -5.52061 |
| Cu | -7.33276 | 35.49179 | -2.17785 |
| Cu | -6.40804 | 39.28102 | -7.25530 |
| Cu | -4.79537 | 26.54493 | -5.45504 |
| Cu | 1.81152 | 30.07880 | -5.27671 |
| Cu | -9.27020 | 31.01960 | -11.06610 |
| Cu | -4.94069 | 24.41109 | -6.67631 |
| Cu | -7.52807 | 39.06212 | -5.03646 |
| Cu | -8.98596 | 39.29904 | -7.04326 |
| Cu | -5.51934 | 37.93930 | -13.09536 |
| Cu | -7.34519 | 34.86217 | -7.06753 |
| Cu | -0.41646 | 36.63370 | -9.20984 |
| Cu | 0.17101 | 31.46789 | -8.85675 |
| Cu | 1.50760 | 28.11431 | -6.85868 |
| Cu | -7.52712 | 36.38768 | -13.39290 |
| Cu | -4.40331 | 28.94706 | -6.73201 |
| Cu | -8.40662 | 33.16900 | -2.01727 |
| Cu | 3.67274 | 28.91804 | -8.85873 |
| Cu | -11.29752 | 38.47063 | -6.87973 |
| Cu | -5.76861 | 33.38565 | -10.54717 |
| Cu | -12.43210 | 37.76580 | -8.91674 |
| Cu | 0.11998 | 35.28475 | -11.30687 |
| Cu | -4.79753 | 35.35342 | -7.69600 |
| Cu | -13.64550 | 29.71927 | -13.08010 |
| Cu | -2.95501 | 37.83670 | -13.57774 |
| Ag | -18.10607 | 28.69278 | -14.93008 |
| Ag | -4.73397 | 32.05244 | -13.77480 |
| Ag | 4.00527 | 29.07902 | -16.31547 |
| Ag | -5.00062 | 22.05188 | -15.94566 |
| Ag | -10.47041 | 21.43475 | -10.79593 |
| Ag | -9.03635 | 30.34192 | -8.44950 |
| Ag | -3.67380 | 26.83787 | -10.16856 |
| Ag | -2.99716 | 33.56815 | -20.36407 |
| Ag | -12.13887 | 21.82511 | -13.00346 |
| Ag | -14.01818 | 27.74853 | -3.78312 |
| Ag | -5.40564 | 21.33806 | -18.73438 |
| Ag | 3.35239 | 37.23212 | -6.23023 |
| Ag | -8.53846 | 28.14464 | -18.37274 |
| Ag | -4.47638 | 33.64079 | -1.05943 |
| Ag | -9.65643 | 26.78817 | -23.73863 |
| Ag | -4.64590 | 38.04568 | -1.54180 |
| Ag | -6.48264 | 30.66458 | -7.04506 |
| Ag | -8.82655 | 30.69626 | -2.78084 |
| Ag | 3.76018 | 30.95141 | -14.06718 |
| Ag | -9.32374 | 40.74313 | -11.02616 |
| Ag | -10.39838 | 29.62838 | -16.77072 |
| Ag | -5.39902 | 25.03838 | -12.24038 |
| Ag | -18.72802 | 32.39174 | -10.63772 |
| Ag | -10.02608 | 39.40393 | -15.86366 |
| Ag | -16.29512 | 36.61119 | -9.53642 |
| Ag | -0.84936 | 20.46991 | -9.06392 |
| Ag | -9.00513 | 35.65313 | -0.22391 |
| Ag | -8.89342 | 41.46328 | -5.08436 |
| Ag | -11.02936 | 22.30645 | -18.01525 |
| Ag | -6.18752 | 27.61228 | -11.12570 |
| Ag | -7.32295 | 20.63943 | -6.21336 |
| Ag | -2.06878 | 25.39346 | -1.38032 |
| Ag | -3.95842 | 42.05814 | -10.42834 |
| Ag | -0.37704 | 34.10332 | -19.26357 |
| Ag | -9.40392 | 25.97259 | -2.31518 |
| Ag | -1.68938 | 28.27473 | -0.17261 |
| Ag | -6.56948 | 30.41472 | -9.78594 |
| Ag | -7.18471 | 37.65621 | -20.20655 |
| Ag | -0.77728 | 33.99257 | -15.26185 |
| Ag | -4.79250 | 19.65524 | -6.53557 |
| Ag | -18.83541 | 33.32538 | -13.37113 |
| Ag | -10.76422 | 31.15102 | -21.40044 |
| Ag | -11.41658 | 27.97372 | -2.41938 |
| Ag | -12.46719 | 24.26682 | -19.61253 |
| Ag | -3.38984 | 29.33134 | -16.47589 |
| Ag | -13.11836 | 26.23368 | -9.79786 |
| Ag | -10.36642 | 37.41564 | -1.72927 |
| Ag | -14.87020 | 38.07836 | -7.38919 |
| Ag | -14.04964 | 36.78425 | -17.17981 |
| Ag | -0.79764 | 30.03137 | -15.51163 |
| Ag | -15.30839 | 31.36293 | -19.18435 |
| Ag | 5.49836 | 31.84137 | -10.74588 |
| Ag | -1.50390 | 20.03775 | -16.14554 |
| Ag | 1.85890 | 32.09945 | -19.14998 |
| Ag | -7.25051 | 26.15826 | -3.96860 |
| Ag | -15.14776 | 33.99756 | -10.18210 |
| Ag | -7.93120 | 30.78514 | -17.52244 |
| Ag | -12.86651 | 24.37526 | -11.97423 |
| Ag | -3.71411 | 20.27249 | -14.17560 |
| Ag | -3.29153 | 25.06366 | -14.06295 |
| Ag | -13.32486 | 29.90375 | -20.44131 |
| Ag | 6.15315 | 32.80359 | -5.96498 |
| Ag | 1.55704 | 27.08540 | -9.17003 |
| Ag | -3.99264 | 40.02603 | -12.49152 |
| Ag | -4.22328 | 26.84013 | -0.02166 |
| Ag | -8.23072 | 25.03246 | -22.17170 |
| Ag | 0.67626 | 24.42249 | -8.34649 |
| Ag | -5.92035 | 24.48006 | -14.93935 |
| Ag | -6.64102 | 28.64652 | -2.66973 |
| Ag | -9.87884 | 30.30311 | -14.01156 |
| Ag | -10.00163 | 26.83011 | -16.20379 |
| Ag | -2.69296 | 26.29375 | -20.37343 |
| Ag | -18.70516 | 31.65625 | -8.06366 |
| Ag | -3.08981 | 21.76446 | -10.17560 |
| Ag | -15.62597 | 27.90697 | -13.83591 |
| Ag | -1.81591 | 27.57102 | -14.81319 |
| Ag | -4.80867 | 34.06935 | 1.53664 |
| Ag | -3.93258 | 29.20889 | 1.29781 |
| Ag | -15.31897 | 26.05627 | -8.07615 |
| Ag | -0.52149 | 32.09089 | -17.49198 |
| Ag | -13.67096 | 38.57684 | -12.68817 |
| Ag | 5.41940 | 32.97018 | -13.20237 |
| Ag | -4.55284 | 22.54963 | -12.99614 |
| Ag | 2.61954 | 27.60613 | -18.32514 |
| Ag | -6.85489 | 22.96556 | -7.83377 |
| Ag | -10.49407 | 41.04136 | -8.60349 |
| Ag | -11.85416 | 23.64544 | -5.82168 |
| Ag | -2.52402 | 34.14698 | -17.57592 |
| Ag | -0.03820 | 26.64320 | -4.50216 |
| Ag | -8.32675 | 40.81875 | -13.72063 |
| Ag | -10.43643 | 31.62443 | -18.67715 |
| Ag | -15.27850 | 25.16698 | -13.11828 |
| Ag | -12.26766 | 33.10297 | -17.09652 |
| Ag | -15.12482 | 37.29913 | -14.76971 |
| Ag | -2.52701 | 38.21396 | -17.14283 |
| Ag | -13.93337 | 30.08586 | -5.63008 |
| Ag | -13.46439 | 28.95495 | -10.34733 |
| Ag | -3.96215 | 27.72576 | -12.92794 |
| Ag | -11.44385 | 28.25089 | -22.12973 |
| Ag | -7.10297 | 26.30834 | -16.67123 |
| Ag | -11.92412 | 24.82644 | -16.81295 |
| Ag | -9.59129 | 25.70155 | -5.10705 |
| Ag | -15.39893 | 32.36115 | -2.62732 |
| Ag | -16.40102 | 32.43517 | -12.17939 |
| Ag | -1.41350 | 24.47408 | -18.54943 |
| Ag | -16.42574 | 31.42848 | -9.47480 |
| Ag | -1.13428 | 39.63983 | -12.62187 |
| Ag | -0.00465 | 26.92127 | -19.34731 |
| Ag | 0.58950 | 28.91396 | -3.22757 |
| Ag | -0.50661 | 23.05242 | -10.54071 |
| Ag | -2.19874 | 41.69756 | -8.22174 |
| Ag | -15.73378 | 27.99892 | -5.88194 |
| Ag | -5.46730 | 25.54023 | -21.34960 |
| Ag | -9.44874 | 23.82612 | -7.42492 |
| Ag | -0.55723 | 39.74116 | -3.17823 |
| Ag | -4.94276 | 20.18123 | -11.41757 |
| Ag | -6.22121 | 30.21520 | -22.25039 |
| Ag | -12.90819 | 26.86964 | -20.08298 |
| Ag | -12.89307 | 30.37291 | -17.87008 |
| Ag | -16.79035 | 29.99806 | -17.05868 |
| Ag | -0.59811 | 36.83063 | -6.53352 |
| Ag | 1.15119 | 38.64280 | -5.16183 |
| Ag | -15.12615 | 34.98714 | -12.82487 |
| Ag | -9.56763 | 23.59774 | -20.05294 |
| Ag | -7.84163 | 21.44635 | -3.68358 |
| Ag | 1.11693 | 29.45028 | -20.04079 |
| Ag | -9.04958 | 24.31985 | -17.32182 |
| Ag | 3.53347 | 31.84571 | -16.76010 |
| Ag | -11.58055 | 33.60288 | -14.33863 |
| Ag | -8.04696 | 28.71000 | -15.63165 |
| Ag | -9.05384 | 39.09326 | -18.65281 |
| Ag | -13.97306 | 32.36266 | -13.61366 |
| Ag | -6.52240 | 38.06270 | -17.60281 |
| Ag | -14.77974 | 38.79814 | -10.03150 |
| Ag | -0.62582 | 24.29419 | -13.32080 |
| Ag | 2.85402 | 25.70867 | -12.79399 |
| Ag | -10.99512 | 39.67355 | -13.17773 |
| Ag | -11.91323 | 28.34622 | -14.75102 |
| Ag | 3.99924 | 28.07511 | -13.42663 |
| Ag | -7.42753 | 31.42235 | -14.79029 |
| Ag | -11.47740 | 21.89396 | -8.08277 |
| Ag | -1.98883 | 19.24526 | -11.23931 |
| Ag | 1.91804 | 23.10050 | -4.63659 |
| Ag | -11.55127 | 41.04277 | -5.91669 |
| Ag | -9.40390 | 27.42939 | -13.51935 |
| Ag | 1.63014 | 35.59172 | -7.57766 |
| Ag | -5.93370 | 35.91401 | -0.08295 |
| Ag | -9.66039 | 20.59125 | -16.26840 |
| Ag | 4.46315 | 33.41684 | -8.37012 |
| Ag | -5.01962 | 26.21540 | -18.53735 |
| Ag | -1.14654 | 29.43503 | -18.31996 |
| Ag | -1.26092 | 30.83851 | 0.87685 |
| Ag | 3.03509 | 24.54669 | -9.89074 |
| Ag | -10.48344 | 23.06877 | -15.21876 |
| Ag | 0.50965 | 20.55193 | -11.69574 |
| Ag | -6.44882 | 28.77796 | 0.15503 |
| Ag | -7.02238 | 28.03519 | -8.40521 |
| Ag | -5.00299 | 41.81838 | -7.82375 |
| Ag | -3.36158 | 31.81714 | 2.38588 |
| Ag | -13.05947 | 32.30640 | -3.95813 |
| Ag | -13.10869 | 22.08803 | -10.31555 |
| Ag | -7.45016 | 23.80280 | -2.25632 |
| Ag | -8.43393 | 23.31218 | -10.11964 |
| Ag | -6.15074 | 20.26268 | -8.86122 |
| Ag | -13.10754 | 40.30844 | -8.00360 |
| Ag | 2.25849 | 31.21106 | -2.60912 |
| Ag | 0.36346 | 27.54631 | -16.67427 |
| Ag | -13.79699 | 26.44761 | -15.26085 |
| Ag | -2.41173 | 26.95459 | -17.67666 |
| Ag | -12.74722 | 32.60869 | -1.19526 |
| Ag | -5.93057 | 28.74667 | -17.45863 |
| Ag | -0.94071 | 39.11544 | -7.96934 |
| Ag | -13.79219 | 25.86126 | -5.74679 |
| Ag | -2.94349 | 22.86894 | -3.09171 |
| Ag | -4.76150 | 37.98176 | -15.56794 |
| Ag | -4.53648 | 36.03927 | -17.57725 |
| Ag | -2.29284 | 35.10644 | -5.05986 |
| Ag | -8.55690 | 30.85517 | 0.05579 |
| Ag | -4.59058 | 40.13457 | -17.33106 |
| Ag | -8.13376 | 25.66979 | -11.59282 |
| Ag | 4.12228 | 36.11209 | -8.61200 |
| Ag | -4.75069 | 24.30578 | -1.27707 |
| Ag | 5.22016 | 27.40706 | -4.80746 |
| Ag | -12.84087 | 32.65601 | -19.84242 |
| Ag | -5.32093 | 31.41439 | -16.76302 |
| Ag | -11.70207 | 35.67383 | -16.13411 |
| Ag | -13.42994 | 28.10529 | -7.41931 |
| Ag | -6.30664 | 23.80024 | -17.67536 |
| Ag | -13.35833 | 27.15085 | -12.57469 |
| Ag | -17.06157 | 32.72657 | -17.43867 |
| Ag | -3.98535 | 23.95753 | -19.65222 |
| Ag | -11.52951 | 31.61467 | -12.30912 |
| Ag | -4.94035 | 33.94400 | -15.99203 |
| Ag | 4.19096 | 29.86927 | -3.87163 |
| Ag | 4.75082 | 34.53135 | -11.10389 |
| Ag | -0.88394 | 21.87264 | -4.58123 |
| Ag | 0.76512 | 21.91328 | -6.86966 |
| Ag | 2.81625 | 26.60793 | -15.63203 |
| Ag | -0.49512 | 41.05922 | -5.90129 |
| Ag | -7.54389 | 23.67070 | -5.29361 |
| Ag | -14.66919 | 31.84564 | -16.40676 |
| Ag | -10.17425 | 37.38712 | -14.21526 |
| Ag | -8.97967 | 29.49908 | -22.91087 |
| Ag | -10.89398 | 26.26813 | -11.35139 |
| Ag | -17.65123 | 33.12691 | -5.94309 |
| Ag | -13.82588 | 22.96132 | -17.51121 |
| Ag | -16.10031 | 30.52948 | -14.34192 |
| Ag | -1.61886 | 26.63748 | -12.17389 |
| Ag | -8.77072 | 20.98713 | -8.62406 |
| Ag | -0.14269 | 22.60132 | -16.72625 |
| Ag | -14.00885 | 34.63312 | -15.51543 |
| Ag | 1.80543 | 25.55940 | -6.17480 |
| Ag | 2.06364 | 34.07125 | -2.39810 |
| Ag | -3.83826 | 28.75847 | -19.28604 |
| Ag | -4.35650 | 21.95947 | -7.80929 |
| Ag | -7.34965 | 33.50037 | 0.30737 |
| Ag | -12.11015 | 35.42523 | -18.91902 |
| Ag | -14.23973 | 25.63991 | -18.07621 |
| Ag | -16.34016 | 35.86121 | -6.75256 |
| Ag | -0.24692 | 35.49233 | -1.16291 |
| Ag | -2.53634 | 25.42593 | -4.29943 |
| Ag | 6.21583 | 30.08095 | -5.83179 |
| Ag | -4.19333 | 19.61369 | -16.79316 |
| Ag | -13.90822 | 35.59588 | -8.28966 |
| Ag | -7.01437 | 27.43845 | -22.81841 |
| Ag | -15.24515 | 33.18819 | -7.64713 |
| Ag | -16.42274 | 35.36610 | -16.63170 |
| Ag | 2.99395 | 28.15336 | -2.02076 |
| Ag | -11.12627 | 30.43381 | -1.10678 |
| Ag | 1.54656 | 24.15436 | -14.92700 |
| Ag | -8.23617 | 21.87887 | -18.41661 |
| Ag | -17.97219 | 27.69038 | -12.24813 |
| Ag | -8.70057 | 28.36914 | -10.56493 |
| Ag | -13.24969 | 38.93567 | -5.34317 |
| Ag | -8.62899 | 25.03056 | -14.52515 |
| Ag | 4.35092 | 32.73329 | -3.74731 |
| Ag | -8.43421 | 30.17549 | -20.22721 |
| Ag | -4.51685 | 26.84935 | -15.71512 |
| Ag | -7.66190 | 25.70610 | -19.43509 |
| Ag | -10.48691 | 38.86157 | -4.39364 |
| Ag | -2.03451 | 27.75240 | -2.90151 |
| Ag | -9.22260 | 34.84433 | -15.07470 |
| Ag | -7.69073 | 32.80637 | -19.42149 |
| Ag | -5.95247 | 31.25342 | 1.38931 |
| Ag | -10.96458 | 28.86807 | -19.42657 |
| Ag | -16.27262 | 37.46770 | -12.08289 |
| Ag | -6.98255 | 28.33660 | -5.46457 |
| Ag | -6.85623 | 41.99171 | -10.12962 |
| Ag | -6.74641 | 23.14408 | -20.44096 |
| Ag | -9.93541 | 23.43146 | -3.56084 |
| Ag | -9.47772 | 36.44891 | -19.80126 |
| Ag | 0.37826 | 21.59889 | -14.38536 |
| Ag | -3.60830 | 31.27558 | -0.25442 |
| Ag | 2.01234 | 36.99957 | -10.19292 |
| Ag | -14.01815 | 31.54177 | -10.90611 |
| Ag | -7.64158 | 34.96563 | -21.33416 |
| Ag | -13.58154 | 30.08316 | -2.41018 |
| Ag | -17.51219 | 34.17071 | -8.56137 |
| Ag | -11.16090 | 28.86669 | -11.98459 |
| Ag | -7.23747 | 23.01877 | -12.77267 |
| Ag | -9.44337 | 27.87915 | -6.92286 |
| Ag | -12.44334 | 21.24825 | -15.86013 |
| Ag | -3.60046 | 30.83439 | -21.25757 |
| Ag | -18.36843 | 29.55311 | -10.07187 |
| Ag | 1.64259 | 29.00807 | -14.54165 |
| Ag | -5.01957 | 30.00410 | -11.99801 |
| Ag | -6.86607 | 26.20678 | -1.00464 |
| Ag | -6.29461 | 31.19614 | -1.42741 |
| Ag | -0.93229 | 31.44878 | -20.27046 |
| Ag | -12.67634 | 34.06303 | -11.66522 |
| Ag | -2.29805 | 36.17952 | -19.45982 |
| Ag | -15.69424 | 26.92492 | -10.87643 |
| Ag | -10.15108 | 23.76816 | -12.44764 |
| Ag | 4.25619 | 24.75618 | -5.12481 |
| Ag | 3.76107 | 31.63883 | -6.18344 |
| Ag | -4.96640 | 35.71964 | -20.42487 |
| Ag | -4.63846 | 26.65395 | -2.83195 |
| Ag | -11.53488 | 30.22234 | -7.04496 |
| Ag | -0.50505 | 36.07758 | -17.09237 |
| Ag | -14.91316 | 36.81266 | -4.51628 |
| Ag | 5.94434 | 28.94839 | -10.25342 |
| Ag | -9.85562 | 38.55869 | -9.41289 |
| Ag | -15.86584 | 28.79805 | -8.86616 |
| Ag | -12.66196 | 38.28512 | -15.25019 |
| Ag | -11.49338 | 25.85350 | -7.34313 |
| Ag | -0.82792 | 19.10805 | -13.70726 |
| Ag | -2.22106 | 20.64075 | -6.63817 |
| Ag | -13.79859 | 34.64219 | -2.83861 |
| Ag | -5.18547 | 33.50578 | -18.77611 |
| Ag | -8.22656 | 32.20175 | -22.01518 |
| Ag | -10.20997 | 33.17231 | -0.23274 |
| Ag | -2.32371 | 22.48289 | -15.11407 |
| Ag | -5.10763 | 24.27280 | -4.03379 |
| Ag | 3.58566 | 29.85874 | -11.42411 |
| Ag | -17.65176 | 34.95037 | -11.37307 |
| Ag | -13.44664 | 23.64807 | -14.68287 |
| Ag | 3.76155 | 30.02045 | -18.88165 |
| Ag | -6.06141 | 41.17909 | -5.42034 |
| Ag | -12.50655 | 36.15266 | -13.52199 |
| Ag | 1.19936 | 35.81259 | -14.83751 |
| Ag | -15.05797 | 24.33000 | -10.20928 |
| Ag | -11.64481 | 27.77773 | -5.26231 |
| Ag | -12.35326 | 36.99994 | -3.46843 |
| Ag | -17.61964 | 26.02511 | -14.33981 |
| Ag | -15.91205 | 29.63258 | -11.55013 |
| Ag | 1.84790 | 22.04641 | -9.41374 |
| Ag | -3.20684 | 36.07895 | 0.08897 |
| Ag | 1.24686 | 25.12115 | -17.46799 |
| Ag | -9.65907 | 34.43128 | -17.88493 |
| Ag | 1.98956 | 23.19267 | -12.17558 |
| Ag | 1.67259 | 38.38452 | -8.01068 |
| Ag | -2.70964 | 31.97089 | -15.64923 |
| Ag | -5.59461 | 32.86553 | -21.48147 |
| Ag | 3.23152 | 33.74840 | -14.60001 |
| Ag | -1.51240 | 28.91058 | -20.90513 |
| Ag | -2.93365 | 24.23167 | -11.49686 |
| Ag | 2.31457 | 25.57361 | -3.33816 |
| Ag | -16.14256 | 30.05842 | -4.04734 |
| Ag | -11.88653 | 25.54949 | -3.77604 |
| Ag | -7.79976 | 22.47019 | -15.50638 |
| Ag | -11.50019 | 37.94332 | -17.92967 |
| Ag | -3.63343 | 24.41430 | -16.86269 |
| Ag | -11.11737 | 28.16292 | -9.03186 |
| Ag | -0.51766 | 24.16929 | -5.87574 |
| Ag | -3.54949 | 19.47515 | -8.96850 |
| Ag | 1.49614 | 35.49503 | -4.84300 |
| Ag | -11.05311 | 25.55206 | -21.65116 |
| Ag | -6.06881 | 39.82581 | -3.06318 |
| Ag | -6.44269 | 25.22856 | -9.43173 |
| Ag | -2.04190 | 37.86255 | -1.54167 |
| Ag | -5.69371 | 30.80933 | -19.47106 |
| Ag | -10.59482 | 26.07388 | -18.99849 |
| Ag | -0.53158 | 36.86804 | -3.70477 |
| Ag | 3.90804 | 27.22017 | -10.77576 |
| Ag | -10.19133 | 33.77675 | -20.60959 |
| Ag | -18.52270 | 31.45795 | -15.47108 |
| Ag | -6.52822 | 28.07598 | -20.18030 |
| Ag | 2.61362 | 35.52260 | -12.44118 |
| Ag | -17.49097 | 35.70792 | -14.02236 |
| Ag | 2.29347 | 34.26962 | -9.97171 |
| Ag | -13.55697 | 23.79435 | -8.03385 |
| Ag | -4.27944 | 28.07166 | -21.86144 |
| Ag | -16.45879 | 33.30662 | -14.73795 |
| Ag | -7.50266 | 38.01663 | -1.28909 |
| Ag | -8.73424 | 39.90571 | -2.80497 |
| Ag | -5.85051 | 42.15086 | -12.52781 |
| Ag | -5.67366 | 40.49435 | -14.65272 |
| Ag | -2.83579 | 39.94323 | -14.95253 |
| Ag | -5.62167 | 21.86281 | -2.05728 |
| Ag | 0.80685 | 29.71921 | -0.72277 |
| Ag | 3.13331 | 23.48082 | -7.41840 |
| Ag | -9.26209 | 21.10477 | -13.51415 |
| Ag | -7.36137 | 40.56799 | -16.67413 |
| Ag | -3.23711 | 41.47061 | -5.59351 |
| Ag | -12.18662 | 39.87489 | -10.62921 |
| Ag | 1.20679 | 31.87015 | -15.06795 |
| Ag | -10.12960 | 35.36888 | -12.37905 |
| Ag | -16.22869 | 27.23889 | -16.51530 |
| Ag | -1.70756 | 23.07721 | -8.02941 |
| Ag | -9.13935 | 27.45612 | -21.12682 |
| Ag | -16.40914 | 30.60334 | -6.83446 |
| Ag | -1.75232 | 21.69996 | -12.59989 |
| Ag | -7.55997 | 38.43573 | -14.94434 |
| Ag | -9.90465 | 32.29640 | -15.95532 |
| Ag | -13.70401 | 36.49669 | -10.87877 |
| Ag | -11.21390 | 24.02591 | -9.68592 |
| Ag | -8.72746 | 38.22995 | -12.07047 |
| Ag | -0.39415 | 33.92409 | -3.52546 |
| Ag | -8.99007 | 30.35559 | -5.50867 |
| Ag | -9.77651 | 21.70476 | -5.75455 |
| Ag | -14.77971 | 28.42840 | -18.52315 |
| Ag | -4.40742 | 38.15574 | -19.32818 |
| Ag | -5.56922 | 29.35020 | -14.67104 |
| Ag | 6.12281 | 28.17489 | -7.63539 |
| Ag | -4.11915 | 29.04911 | -1.54857 |
| Ag | -14.56860 | 34.10491 | -18.32396 |
| Ag | -0.73719 | 38.00994 | -15.13418 |
| Ag | -6.97339 | 20.02755 | -16.69333 |
| Ag | -16.38860 | 34.64244 | -4.06084 |
| Ag | 5.39834 | 30.79013 | -8.22766 |
| Ag | -8.86197 | 28.36619 | -1.20857 |
| Ag | -18.47108 | 30.49745 | -12.64949 |
| Ag | 1.65918 | 34.02602 | -16.98853 |
| Ag | -1.97860 | 38.91837 | -5.36113 |
| Ag | 5.91696 | 30.05862 | -12.73890 |
| Ag | -3.29128 | 39.92892 | -3.20789 |
| Ag | -2.64538 | 21.94829 | -17.93134 |
| Ag | -7.78433 | 41.46630 | -7.62870 |
| Ag | -15.66076 | 24.45133 | -16.03671 |
| Ag | -7.06105 | 32.03260 | -12.03138 |
| Ag | 0.24303 | 39.16850 | -10.37105 |
| Ag | -9.16722 | 36.88982 | -16.91529 |
| Ag | 0.85002 | 37.67961 | -12.72772 |
| Ag | -7.73910 | 20.63479 | -11.20273 |
| Ag | -9.22727 | 26.09235 | -8.92120 |
| Ag | -6.42514 | 20.51593 | -13.89053 |
| Ag | -6.83304 | 33.99667 | -13.87613 |
| Ag | -6.75846 | 26.85924 | -13.81948 |
| Ag | -5.84807 | 22.63192 | -10.44742 |
| Ag | 0.15038 | 32.36710 | -1.19481 |
| Ag | -5.14794 | 21.88432 | -5.11677 |
| Ag | -3.16285 | 31.45369 | -18.50987 |
| Ag | 0.65895 | 25.54702 | -11.13807 |
| Ag | 3.97819 | 26.55928 | -7.63864 |
| Ag | -3.73148 | 39.45679 | -7.66646 |
| Ag | -0.93761 | 25.14254 | -15.84567 |
| Ag | -2.01795 | 33.42764 | 0.25441 |
| Ag | 3.87435 | 34.60298 | -5.89581 |
| Ag | -7.61541 | 29.40941 | -12.78015 |
| Ag | -11.44754 | 25.68450 | -14.16778 |
| Ag | -0.22275 | 24.04983 | -3.12499 |
| Ag | 0.41365 | 26.74550 | -1.66665 |
| Ag | 1.48340 | 30.07324 | -17.29119 |
| Ag | -11.27814 | 30.20761 | -3.93941 |
